# Eating for Two? Protocol of an Exploratory Survey and Experimental Study on Social Norms and Norm-Based Messages Influencing European Pregnant and Non-pregnant Women’s Eating Behavior

**DOI:** 10.3389/fpsyg.2018.00658

**Published:** 2018-05-08

**Authors:** Kirsten E. Bevelander, Katharina Herte, Catherine Kakoulakis, Inés Sanguino, Anna-Lena Tebbe, Markus R. Tünte

**Affiliations:** ^1^Communication Science, Behavioural Science Institute, Radboud University, Nijmegen, Netherlands; ^2^Department of Social, Health and Organisational Psychology, Utrecht University, Utrecht, Netherlands; ^3^Department of Psychology, University of Nicosia, Nicosia, Cyprus; ^4^Department of Psychology, King’s College London, London, United Kingdom; ^5^Department of Psychology, University of Mannheim, Mannheim, Germany; ^6^Faculty of Psychology, University of Vienna, Vienna, Austria

**Keywords:** social norms, descriptive and injunctive messages, pregnant women, social norm messages, snacking behavior, sugar-sweetened beverages, behavioral nutrition

## Abstract

The social context is an important factor underlying unhealthy eating behavior and the development of inappropriate weight gain. Evidence is accumulating that powerful social influences can also be used as a tool to impact people’s eating behavior in a positive manner. Social norm-based messages have potential to steer people in making healthier food choices. The research field on nutritional social norms is still emerging and more research is needed to gain insights into why some people adhere to social norms whereas others do not. There are indications stemming from empirical studies on social eating behavior that this may be due to ingratiation purposes and uncertainty reduction. That is, people match their eating behavior to that of the norm set by their eating companion(s) in order to blend in and be part of the group. In this project, we explore nutritional social norms among pregnant women. This population is particularly interesting because they are often subject to unsolicited advice and experience social pressure from their environment. In addition, their pregnancy affects their body composition, eating pattern, and psychosocial status. Pregnancy provides an important window of opportunity to impact health of pregnant women and their child. Nevertheless, the field of nutritional social norms among pregnant women is understudied and more knowledge is needed on whether pregnant women use guidelines from their social environment for their own eating behavior. In this project we aim to fill this research gap by means of an exploratory survey (Study 1) assessing information about social expectations, (mis)perceived social norms and the role of different reference groups such as other pregnant women, family, and friends. In addition, we conduct an online experiment (Study 2) testing to what extent pregnant women are susceptible to social norm-based messages compared to non-pregnant women. Moreover, possible moderators are explored which might impact women’s susceptibility to social norms as well as cultural aspects that co-determine which social norms and guidelines exist. The project’s findings could help design effective intervention messages in promoting healthy eating behavior specifically targeted to European pregnant women.

## Introduction

There is substantial literature examining the general impact of the social environment on behavior (Cialdini et al., 1991), and evidence is accumulating that social influences on eating behavior are powerful as well (Christakis and Fowler, 2007; Salvy et al., 2012; Cruwys et al., 2015). Several methodological approaches have been used to investigate social influence on eating. Besides correlational studies, observational social eating studies have shown that people conform their eating behavior to social norms set by others, and that people converge upon an eating norm when eating together. For example, people who ate with a ‘confederate’ instructed to eat a certain type or amount of food were found to imitate or adjust their food choices and intake to that of their instructed eating companion (Salvy et al., 2012; Cruwys et al., 2015; Higgs, 2015). To date, the social context is increasingly recognized as an important factor underlying the development of inappropriate weight gain (Christakis and Fowler, 2007). Even though it is completely normal to gain weight for pregnant women, up to 40% gains more weight than recommended by health guidelines while this can have lifelong detrimental effects on both mother and child (Thangaratinam and Jolly, 2010). Surprisingly, the field of nutritional social norms among pregnant women is understudied (Gardner et al., 2012; Hutchinson et al., 2017). This population is of interest in particular, because pregnancy is a crucial period in life at which women may be more or less susceptible to social norms and dietary change than usual (Campbell et al., 2011). That is, pregnant women often experience that they receive unsolicited advice from everyone who knows or sees they are pregnant, and they have to deal with many implicit as well as explicit rules they are subject to by their pregnancy (Root and Browner, 2001; Graham et al., 2013). Moreover, they may feel ‘allowed’ to consume more unhealthy foods as their body composition changes and weight gain is more socially acceptable, despite being aware that their unborn child benefits from healthy eating (Campbell et al., 2011). Thus, pregnancy can provide women a reason or an excuse to change their diets for better or worse. The overarching goal of this project is to gain more insight into whether and how social norms play a role in pregnant women’s eating behavior.

To our knowledge, literature is limited to few correlational studies examining social norms among pregnant women. Health norms during pregnancy in general were brought to attention in the 1970s in a study on smoking, drinking, eating, and physical activity (Barić and MacArthur, 1977). However, only two recent studies have further investigated the influence of the social environment on dietary intentions and self-reported food intake (Gardner et al., 2012; Hutchinson et al., 2017). A study by Gardner et al. (2012) investigated whether *subjective norms* (i.e., anticipated social approval from the social environment to eat healthy) predicted healthy eating intentions among pregnant women in the United Kingdom. They found positive correlations between approval of eating behavior and family and health care expectations, although there was no direct association between social approval and healthy eating intentions. In a similar manner, a study by Hutchinson et al. (2017) among Australian pregnant women found that endorsement of healthy eating by others was unrelated to dietary intake. The authors speculated that pregnant women may differ from the general population in terms of susceptibility to social influences, because they are more concerned with changing their entire health behavior (e.g., alcohol consumption and smoking) for the benefit of their baby’s health regardless of others’ views. Additionally, they noted that more knowledge is needed on whether social influence depends on which reference group conveys the norm (e.g., partner, mother, family, pregnant, and non-pregnant friends) as well as whether conveyed norms need to be related to pregnancy. Importantly, both studies concluded that further research is warranted to fully understand the influence of social norms on pregnant women’s eating behavior given that food intake usually takes place in social contexts (Gardner et al., 2012; Hutchinson et al., 2017).

To sum up, social norms may determine to a large extent what ‘normal’ as well as ‘acceptable’ eating behavior is for pregnant women. In this project, we examine the influence of social norms on pregnant women’s eating behavior in a systematic approach. First, we aim to generate and refine hypotheses to give direction to future research (Study 1). Based on previous literature, nutritional social norms, and possible moderators are explored by means of an online survey. We then investigate pregnant women’s susceptibility to social norm-based messages in a commonly used experimental research design (Study 2), based on existing literature and the determinants that emerge from Study 1.

### Theoretical Framework Study 1: Exploring Pregnant Women’s Susceptibility to Social Eating Behavior

#### Social Norm Perceptions

The first aim of Study 1 is to examine the perception of social norms among pregnant women. Perceptions and beliefs about the ‘normal’ eating behavior of others influence people’s own behavior. For example, a person’s perception about what influential others or the majority of peers do (i.e., perceived *descriptive* peer norm) may be an important factor in determining what people choose to eat and drink. Nonetheless, perceived peer norms do not always match the actual norm, which can lead to a ‘false consensus’ effect. That is, people direct their behavior to a false and *misperceived* norm. Ample studies in normative misperceptions have shown that people often overestimate the unhealthy behaviors peers do (e.g., alcohol, tobacco and substance use, and risky sexual behavior) (Haug et al., 2011; Bertholet et al., 2013; Lewis et al., 2014) whereas healthy behaviors tend to be underestimated (e.g., condom use and seat belt use) (Lewis et al., 2014; Litt et al., 2014). Likewise, studies on consumption behavior have found that people overestimated peers’ unhealthy food and drink intake but underestimated their fruit and vegetable consumption (Lally et al., 2011). As directing one’s behavior toward a false norm can be harmful and requires correction, gaining insight into misperceived norms is an important topic of investigation. We expect to find similar tendencies among pregnant women and therefore (H1) hypothesize that pregnant women tend to overestimate unhealthy consumption while underestimating healthy consumption norms.

#### Susceptibility to Social Norms

The second aim of Study 1 is to explore factors that may influence how susceptible pregnant women are to social norms. An important topic of investigation is which reference group women refer to concerning their own eating behavior since the beginning of their pregnancy. It has been shown that perceived shared group membership (i.e., others being similar to the self) plays a role in the degree to which people conform their behavior to others (Pachucki et al., 2011; Robinson et al., 2014b). For example, researchers have found that when an out-group member (dissimilar to the self) provides a healthy eating norm, reactance is triggered by which people eat more unhealthily and vice versa (Oyserman et al., 2007; Berger and Rand, 2008; Stok et al., 2012). More knowledge is needed on whether (and when) pregnant women regard other pregnant women, or their family and friends as their in-group. One could argue that exposure to pregnancy-related norms occurs only during 9 months, which is a relatively brief period compared to general normative influences that are accumulated over a lifetime (Hutchinson et al., 2017). Therefore, Study 1 explores which reference group affects pregnant women’s eating behavior (e.g., when sharing a meal with non-pregnant others) utilizing a primarily qualitative approach. It also addresses whether similarity feelings depend on the duration and visibility of pregnancy which can make women feel more or less similar to particular reference groups.

In addition to insights on shared group membership, research has shown that conforming to social norms depends on the social bond between people and that this seems to be motivated by the desire to affiliate and reduce uncertainty (Cruwys et al., 2015). For example, a cold and distant social interaction during dinner led women to direct their eating behavior more toward that of a cold acting eating companion than when the social interaction was warm and friendly, which is believed to reflect an ingratiation attempt (Hermans et al., 2009). Other studies have focused on the role of empathy, self-esteem, body-esteem, and sociotrophy to scrutinize which underlying mechanisms are at play in social modeling behavior (Robinson et al., 2011; Exline et al., 2012; Hirata et al., 2015). For example, in an experimental study asking female dyads to complete a problem solving task together while having access to food, the degree of matching food intake was associated with their empathy and self-esteem (Robinson et al., 2011). That is, women with lower self-esteem were found to match their intake more than those with higher self-esteem. In a study of Exline et al. (2012), sociotropy predicted people’s attempts to match their companion’s eating with their own to make their companion feel comfortable. In addition, it predicted more personal distress related to social pressure by eating more. Although findings are mixed, the general pattern indicated that people tend to conform their consumption behavior more when they feel uncertain, want to please others or fear to be socially excluded (Cruwys et al., 2015).

With regard to pregnant women, research has shown that they are confronted with changes in their psychosocial status (e.g., anxiety, stress, depression, and self-esteem) (Hickey et al., 1995) that can affect their well-being and weight gain during pregnancy (DiPietro et al., 2003). Based on social eating literature (Cruwys et al., 2015), it is plausible that these factors also play a role in pregnant women’s reactions to social normative information. For example, research has shown that self-esteem plays a role in the perception and need of feeling socially accepted (Baumeister and Leary, 1995). That is, people with higher self-esteem tend to worry less about how they are seen by others and conform less to other’s behavior (Leary and Baumeister, 2000). Interestingly, pregnancy can be seen as a time when being large is socially acceptable (Campbell et al., 2011) and therefore can give a sense of confidence. In addition, it has been suggested that pregnant women who are preoccupied with the health of their child may feel less uncertain because they are more likely to strictly follow dietary guidelines (Hutchinson et al., 2017). In turn, this might make them less susceptible for specific food norms from their social environment (Hutchinson et al., 2017). In contrast, pregnant women were also found to experience less self-confidence because they feel less physically attractive, subject to public’s opinions and limited in their activities (Campbell et al., 2011; Graham et al., 2013). Given that this project is the first that investigates underlying mechanisms of social norm influences among pregnant women, we explore whether and how factors linked to ingratiation and uncertainty reduction (e.g., self-esteem, need to belong and perceived social support or sabotage) play a role in social eating behavior among pregnant women. We (H2) postulate that factors related to affiliation purposes and uncertainty reduction also affect the susceptibility to social norms in pregnant women.

Further, and although not linked directly to social eating behavior, studies have found other factors influencing pregnant women’s health behaviors in general such as mindful eating, anxiety, self-regulation, and impulsivity (Rofé et al., 1993; Hickey et al., 1995; Hutchinson et al., 2017). These factors may moderate the degree of pregnant women’s responsiveness to social norms. For example, their self-control may be increased when they are determined to eat healthy for the benefit of their baby resulting in disregard for influences from their social environment. Given that more knowledge is necessary on whether psychological determinants affect pregnant women’s susceptibility to social influences, we also explore the role of above mentioned psychological factors outside the ingratiation theme in a qualitative manner.

In conclusion, a deeper understanding of social, personal, and psychological factors underlying pregnant woman’s eating behavior is needed. By means of an exploratory survey, Study 1 assesses information about (mis)perceived social norms and social expectations, and the role of different reference groups such as other pregnant women, family, and friends (Campbell et al., 2011). Moreover, we aim to identify underlying mechanisms (e.g., misperceived expectations or sensitivity to social sanctions) that might explain their behavior and whether health considerations may decrease social susceptibility (Hutchinson et al., 2017).

### Theoretical Framework Study 2: Experimental Study of Social Norm-Based Messages on Food Choice

#### Social Norm-Based Messages

Next to the empirical social eating literature (Cruwys et al., 2015), people have been found to adhere to norms in situations where individuals were merely exposed to *written* information about what other people did. Typical experimental studies on written norms expose people to information about people’s eating behavior and through social norm messages (e.g., by exposure to a poster message in an evaluation task or to information about what prior participants had eating during a task or test) (Robinson et al., 2013, 2014b; Stok et al., 2014). Social norm-based messages have succeeded in changing intentions and behaviors unrelated to health (e.g., pro-environmental behavior) (Goldstein et al., 2008; van der Linden, 2015), but evidence is accumulating that it could be applied in the eating domain as well (Stok et al., 2012; Robinson et al., 2014b; Higgs, 2015; Robinson, 2015). In general, norm-based messages suggesting that others ate large portions of food were associated with increased food intake, and vice versa. In addition, information about food choice norms was found to influence the consumption of unhealthy snack food as well as healthy snacks such as fruit and vegetables (Robinson et al., 2014b; Robinson, 2015). These findings suggest that so-called ‘social norm-based’ messages have potential to steer people in making healthy food choices.

Research on social norm-based messages related to eating behavior has focused mainly on two types of messages, namely *descriptive* and *injunctive* messages (Burger et al., 2010; Mollen et al., 2013; Robinson et al., 2013, 2014b; Stok et al., 2014; Robinson, 2015). Descriptive messages provide general information and describe what is the ‘normal’ consumption of (the majority of) others, whereas injunctive messages proscribe what is socially approved off and is found to be the ‘appropriate’ consumption according to others (i.e., how others want you to behave). There is a limited number of studies testing both descriptive and injunctive norm-based messages (versus a health message or no-message control condition). These studies have shown mixed findings. For example, a correlational study on fruit intake found that compared to a control group, individuals reported having taken more fruits after being exposed to a descriptive norm but not to an injunctive norm message (Stok et al., 2014). Another study testing a healthy descriptive and injunctive norm and an unhealthy descriptive norm also found that compared to a control group, a healthy descriptive message led to more healthy choices whereas a healthy injunctive norm did not (Mollen et al., 2013). None of the norm messages affected unhealthy food choices in this study (Mollen et al., 2013), whereas another study testing a descriptive norm on junk food intake found opposite results (Robinson et al., 2013). A descriptive norm message reduced junk food intake compared to a control message; however, it did not reduce intake any more than a health message (Robinson et al., 2013). A study that compared a descriptive healthy norm with a healthy message did find a significant effect for the norm over a health message (Robinson et al., 2014a). Overall, descriptive norm messages seemed to have the biggest impact on healthy food intake (Burger et al., 2010; Mollen et al., 2013; Robinson et al., 2013; Stok et al., 2014). As an explanation, it has been argued that a descriptive type of message does not threaten people’s sense of freedom compared to an injunctive norm (Stok et al., 2014). That is, telling people explicitly that they should (not) do something (i.e., injunctive norm), may lead to a dismissal of the message or might even provoke an opposite response (‘boomerang effect’ or ‘reactance’) (Knowles and Linn, 2004; Brehm and Brehm, 2013). Remarkably, none of the norm-based message studies have investigated whether psychosocial determinants influence people’s susceptibility.

Study 2 advances knowledge in the emerging field of research on social norm-based messages on eating behavior by focusing on two parts. In part A, we investigate general differences between pregnant and non-pregnant women after exposure to norm-based messages. We (H3) speculate that a descriptive norm message has a positive effect on pregnant as well as non-pregnant women’s healthy food choice compared to a control condition. Given that pregnant women are subject to unsolicited advice, rules and regulations from society (Root and Browner, 2001), we (H4) hypothesize that exposure to an injunctive norm-based message causes a reactance effect on food choice (meaning that women will choose more unhealthy foods) compared to a descriptive norm message and a control condition. We expect this effect to be stronger for pregnant than non-pregnant women. Part B explores moderating variables of social norm-based messages on food choice in the pregnant and non-pregnant women samples separately, based on the outcomes of Study 1 and social eating literature. Similar to Study 1, we explore which factors play a role in the susceptibility to social norms.

#### Societal Relevance

Lifestyle and dietary habits of pregnant women have lifelong effects on themselves and their child’s weight and health (Birdsall et al., 2009). Interventions and educational activities generally aim to inform pregnant women about the harm of smoking, alcohol consumption, and drug use (Johnson et al., 1987; Lumley et al., 2009; Stade et al., 2009; Nilsen, 2010). Regarding nutrition, most advice is focused on preventing women from suffering deficiencies in micronutrient requirements (e.g., vitamins and folic acid) or eating high risk foods (e.g., unpasteurized milk and soft cheese or raw fish and meat) causing listerial infection or toxoplasmosis that can affect fetal and child development (Ray and Laskin, 1999; Willers et al., 2007; Guelinckx et al., 2008; Janakiraman, 2008; Paquet et al., 2013). An important topic that has received less attention but could nonetheless be fruitful in terms of intervention is the prevention of excess weight gain during pregnancy (Cogswell et al., 1999; Strychar et al., 2000). It is increasingly recognized that inappropriate weight gain during pregnancy has persisting effects on child adiposity, cognitive development, blood pressure, and atopic disease as well as post-partum weight retention among mothers (Birdsall et al., 2009; Graham et al., 2013). Despite the short and long term neonatal and maternal benefits of an appropriate diet during pregnancy, 20–40% of pregnant woman in Europe gain more weight than recommended by health guidelines (Thangaratinam and Jolly, 2010). As explained above, social norms can motivate and direct a person’s behavior, because they are linked to social sanctions and rewards for (non)conformity, and social expectations. It is suggested that when one wants to influence behavior permanently, social norms need to be changed first (Barić and MacArthur, 1977). This project is the first to investigate whether social norms can be used as an effective method to impact dietary intake of pregnant women for the benefit of both mother and child.

## Study 1 – Online Survey: Exploring Pregnant Women’s Susceptibility to Social Eating Behavior

### Stepwise Procedures

#### Translation of Materials

For Study 1 (and 2) the authors translate all materials from English to their countries’ official language (i.e., forward translation). An English native speaker with a proficiency level in the target language translates the survey from the target language to English (i.e., back translation). The translations are reviewed and compared, discussing disagreements until consensus is reached. In both studies, pilot tests are conducted to receive feedback regarding the clarity and length of the materials and the presentation of the questionnaire measurements.

#### Design and Participants

The general aim of Study 1 is to explore (mis)perceived social norms and expectations of different reference groups together with potential underlying mechanisms that influence pregnant women’s (un)healthy snacking and drinking behavior. We use an exploratory mixed method approach (i.e., qualitative as well as quantitative) to collect information via an anonymous online survey with open-ended and closed questions. Results will be used to extract moderator variables for Study 2.

The study will take place between March and May 2018. Women between 18 and 40 years old with uncomplicated singleton pregnancies are eligible to participate in the study. Given that the study aim is primarily exploratory, a conservative *a priori* power analysis (G^∗^Power 3.1.9.2) for the regression analysis was performed accounting for seven predictors and a moderate effect size (two-tailed, f^2^ = 0.01; power 0.95, α = 0.05). This resulted in a total sample size of at least *N* = 132 participants. Taken into account an attrition rate of 15–20%, we aim to recruit at least 25 participants per country.

Participants are recruited by a purposive sampling method in six European countries (i.e., Netherlands, Germany, United Kingdom, Spain, Austria, and Cyprus/Greece) (Etikan et al., 2016). Advertisements are placed at typical locations where pregnant women come (e.g., midwife practices, medical offices, pregnancy yoga, or swimming classes) and on online platforms (e.g., at parenting and pregnancy forum, specific Facebook groups, and online newsletters). The advertisements fully explain the aim of the study and invite pregnant women to participate in the online study. Before starting the questionnaire, they can read additional information about the study aim, procedure, and context to ensure transparency and allow for proper consideration of participation. The participants are also informed about the anonymity and confidentiality of their answers and the right to withdraw from the study at any stage.

### Materials and Equipment

#### Measures

Participants fill out open and closed-ended online questionnaires using online survey software (Qualtrics), covering demographic and pregnancy-related information, social norms, snacking and drinking behavior and psychological factors. All survey measurements are existing validated questionnaires, translated into the appropriate language. Some questionnaire items are tailored to identify specific behaviors among pregnant women. All measurements are described in detail in the following paragraphs.

##### Demographics

Self-reported demographics are assessed by asking for participant’s age, gender, nationality, height and weight before pregnancy, weight gain, level of education and socio-economic status. To ensure that cultural differences in the study are accurate, participants are asked since when they have lived in their current place of residence. Participant’s Body Mass Index (BMI) before pregnancy will be calculated using the standard formula weight [kg]/height^2^ [m].

##### Pregnancy related measurements

Pregnancy related items that potentially affect women’s diet or susceptibility to social norms are assessed, such as duration of pregnancy, singleton or twin pregnancy, parity and experience with miscarriages. In addition, pregnancy ailments are administered on a six-point scale ranging from ‘*Never*’ (1) – ‘*Always’* (6), such as nausea, stomach acid, loss or increase of appetite, constipation, tiredness, etc. (Hutchinson et al., 2017).

##### Eating behavior

Although pregnancy can have influence on a women’s entire diet, this project focuses on snack consumption and beverage intake, specifically. An increased consumption of palatable high-sugary and -fat snack foods (e.g., cakes, biscuits, and crisps) and sugar-sweetened beverages (SSB’s) has been found to contribute to inappropriate weight gain (Olafsdottir et al., 2005; Crozier et al., 2009; Graham et al., 2013). Meeting recommendations related to sugar intake during pregnancy reduces complications and supports appropriate weight gain, optimal fetal growth, and childhood development resulting in an improved health for both mother and her newborn (Birdsall et al., 2009).

*Self-reported fruit consumption.* It is assessed by asking participants to report their fruit consumption of the past 2 days. A list of 26 commonly consumed fruits (for every country involved) is provided. Participants indicate the type and amount of fruit they had consumed (in handfuls for small fruits such as raspberries and in pieces for larger fruits such as apples). In addition, three ‘*other’* options are provided enabling participants to add fruits to the list. In line with previous research, consumption is calculated by computing the total amount of portions of fruit consumed (Verkooijen et al., 2015). For example, two or three pieces of smaller fruits (e.g., prunes) equal one portion normal-sized fruits (e.g., apple) whereas parts of large fruits (e.g., melon) count as one portion. Average daily consumption is calculated by dividing the number by 2 days.

*Self-reported snack consumption.* Similar to the fruit assessment procedure, a list with 13 unhealthy snacks is presented including small or large cookies, sweets, chocolates, warm snacks, etc. The total number of unhealthy snacks will be calculated in the same way as fruit consumption and divided by 2 days (Verkooijen et al., 2015).

*Beverage consumption.* Beverages are assessed by asking the number of glasses (equaling cans, bottles, and packages of 220 ml) participants drank during the past 2 days. Administered drinks are (sparkling) water, dairy products, sugar-sweetened soft drinks, artificially sweetened (i.e., diet) soft drinks, fruit-flavored drinks (i.e., lemonade), fruit juice, energy drinks and (sweetened) tea and coffee. Response categories range from ‘*zero glasses per day’* (0) to ‘*five glasses per day’* (5) (Smit et al., 2016). Drinks are classified as sugar-sweetened and energy-dense ‘unhealthy’ or low sugary and low-energy dense ‘healthy’ (Briefel et al., 2009).

##### Normative information and influence of the social environment

To develop and refine hypotheses for future studies, participants’ social norm expectations and (mis)perceptions as well as the role of different reference groups such as other pregnant women, family, and friends that convey the social norms are explored. In addition, the impact of the social environment is assessed by asking about the women’s social surroundings.

*Actual norms.* The actual norms are calculated by the daily average number of servings of fruits and snacks, and glasses of healthy and unhealthy beverages consumed.

*Identification with norm referent group.* To assess influential individuals in pregnant women’s social surrounding, participants rate the extent to which they identify with different reference groups such as family, partner, and friends (Stok et al., 2012) (e.g., *I feel a strong connection to other pregnant women*), on a six-point scale ranging from ‘*not at all’* (1) to ‘*very much’* (6).

*Perceived descriptive norms.* Participants are asked to estimate how many servings of fruit, snacks and glasses of SSB’s they think other pregnant women generally eat and drink per day. Additionally, the same question is asked for the reference group they indicate to be most important (e.g., family, partner, or friends).

*Misperceived norms.* Misperceived norms are calculated by subtracting the actual mean number of fruits, snacks, and drinks from the perceived actual mean number (for pregnant and other reference group, separately). A lower score indicates that participants underestimated their consumption, a higher score indicates an overestimation of the norm respectively.

*Perceived injunctive norms.* Injunctive norms are operationalized by calculating how many servings of fruit, snacks, and drinks participants think other pregnant and non-pregnant women approve of and think they should consume. The same question is asked for the reference group they indicate to be most important.

*Social expectations.* In addition to the perceived injunctive norm, participants are asked whether they think that it is generally expected by other pregnant women that a pregnant woman should modify her diet (Barić and MacArthur, 1977). This is also assessed for their most important reference group.

*Social support and sabotage.* Social support from friends and family for eating healthy is measured by selected items of the Friend and Family Support for Healthy Eating Habits scale (Sallis et al., 1987). Participants are asked ‘*How often…’* they feel that friends or family support or sabotage their healthy eating by answering options on a six-point scale ranging from ‘*Never*’ (1) – ‘*Always*’ (6). Example questions are *‘…does your family encourage you to eat healthy foods?’* and *‘… do friends eat unhealthy foods in front of you?*’ Participants are given examples of healthy (e.g., low-fat and low-sugar foods illustrated with example products) and unhealthy foods before starting the questionnaire. Originally, the scale contains items about low-fat and low-sugar foods separately, but we combined them into one ‘unhealthy’ item to simplify the question and shorten the list of items.

##### Ingratiation and uncertainty reduction

The extent to which pregnant women are susceptible to social normative influences may be determined by factors related to ingratiation purposes and uncertainty reduction (Cruwys et al., 2015). The following measures are assessed to explore whether these factors play a role among pregnant women as well.

*Self-esteem.* Social and appearance self-esteem is measured by two subscales of the State Self-Esteem Scale (SSES) (Heatherton and Polivy, 1991). The SSES is a 20-item questionnaire measuring both positive and negative thoughts about oneself related to social and appearance self-esteem. Items are framed as ‘*How often…*’ participants feel worthy or not about themselves [e.g., ‘*How often are you feeling unattractive?’* (appearance) or ‘…*are you concerned about the impression that you make?’* (social)] with answering categories ranging from ‘*Never*’ (1) to ‘*Always*’ (6).

*Fear of negative evaluation.* The fear and distress of being evaluated unfavorably by others in a social situation is measured by the 12-item Brief Fear of Negative Evaluation Scale (BFNE) (Leary, 1983). Items are prefaced with ‘*How often…*’ participants feel distress or social anxiety with answering categories ranging from ‘*Never*’ (1) to ‘*Always*’ (6) (e.g., ‘*How often are you afraid of other people noticing your shortcomings?*’ or ‘…*do you worry that you will say or do the wrong things?*’).

*Health benefits.* Pregnant women’s susceptibility to social norms may be influenced by how preoccupied women are with their own and their baby’s health (Hutchinson et al., 2017). The Nutrition Benefit scale is adapted from previous research (Tiedje et al., 1992) and participants rate how strongly they agree with statement such as ‘*If I don’t eat healthy, there could be something wrong with my baby*’ and ‘*Good nutrition during pregnancy will prevent me from gaining a lot of weight*’ on a six-point scale ranging from ‘*Not at all*’ (1) to ‘*Very much*’ (6). In addition, we use the Figure Rating Scale which depicts nine silhouettes of female adult body figures ranging from very thin (1) to obese (9) (Stunkard et al., 1983). Participants indicate their figure before pregnancy and their desired figure 6 months after pregnancy. The difference score between the desired and actual figure provides an indication of women’s preoccupation about their weight and appearance.

##### Social desirability bias

Although this research is related to social desirable behavior, we also want to take a possible social desirability bias into account. This refers to the tendency of participants to answer in a particular way that will be viewed favorably by others. Therefore, the short Marlowe-Crowne-Social Desirability Scale (SDS) is used to measure socially desirable responses (Reynolds, 1982; Loo and Thorpe, 2000). It consists of 10 items prefaced by ‘*How often…*’ (e.g., ‘…*do you like to gossip?*’ and ‘…*are you willing to admit it when you make a mistake?*’) with answering categories ranging from ‘*Never*’ (1) – ‘*Always*’ (6). The SDS can detect a form of over-reporting ‘good behavior’ or under-reporting ‘bad’ or undesirable behavior.

##### Open-ended survey items

To collect more in-depth information about social normative behavior and pregnant women’s susceptibility to social norms, open-ended questions are administered throughout the survey (Root and Browner, 2001). Apart from filling in demographic and pregnancy related information, women are asked whether, how and why pregnancy changed their life in general and in relation to their diet, specifically. In addition, we explore which people (i.e., reference group) may have an important influence on pregnant women’s diet and choices (e.g., ‘*Who in addition to your physician or nurse do you turn to for information or advice during your pregnancy?*’). Next, other factors that have been raised by scarce literature on pregnant women’s health behaviors are presented (i.e., mindful eating, anxiety, self-control, and impulsivity) (Rofé et al., 1993; Springer et al., 1994; Hickey et al., 1995; Hutchinson et al., 2017). Participants are asked to elaborate on whether and why (not) they think that they influence their own susceptibility to social norms. The factors are clarified before they are presented to the participants. Finally, the participants are invited to think of additional factors concerning the role of social norms and susceptibility on their eating behavior.

### Proposed Analysis and Anticipated Results

Aim of the analyses are generating insights into (mis)perceived social norms, reference groups and underlying mechanisms of susceptibility to social norms among pregnant women. Results from these exploratory analyses serve to define and test specific hypotheses for Study 2 in an experimental setting.

The methodological principles of the qualitative data part are based on the grounded theory approach, which is a systematic procedure for building new theory (Corbin and Strauss, 2008). Qualitative data stems from responses to the open-ended survey items. The responses are coded by multiple researchers (i.e., the authors) to minimize coding bias and increase reliability of the coding procedure. Through the coding process, researchers create meaningful labels for participants answers. The procedure allows to make sense of data in a flexible and iterative process in which the analysis procedure goes back and forth until no new coding categories occur and ‘theoretical saturation’ is reached. English transcriptions are analyzed using thematic content analyses (Braun and Clarke, 2006). Data are analyzed (MAXQDA software package) conducting (1) open coding followed by (2) axial coding (i.e., making salient and subcategories) and (3) selective coding (i.e., integrating categories into theoretical concepts) (Corbin and Strauss, 2008; Boeije, 2010). All core categories and identified relationships are documented in a coding dictionary. (1) After familiarizing with the data, the coders generate initial codes and place them into top-level ‘labels’ or ‘themes’ (e.g., home environment, psychosocial factors). Coders compare their individual codes and discuss them until consensus is reached. (2) Axial coding is used to create main and subcategories of the codes. This is done by identifying relationships between and across the themes while examining recurring phenomena, actions, and interactions. The coders discuss their categorizations and after reaching consensus, the identified themes can be further divided into second-level categories. (3) The final step is to integrate the core categories into theory. The themes and subcategories that emerge can provide insight for future directions of research (Corbin and Strauss, 2008; Boeije, 2010). For example, they may support or counter our assumption that ingratiation plays a role in being susceptible for social influence, or point to a different factor that emerged from the open-ended items which needs to be further investigated. In addition, it is possible that pregnancy related items (e.g., the duration of pregnancy and visibility/body figure) appear to play an important role in being susceptible to social norms. The top-level categories and their determinants are also compared between countries to investigate cross-cultural differences.

For the quantitative data analyses, scale reliability and construct validity is assessed by factor analysis according to standard procedures. To test whether participants overestimate the unhealthy and underestimate the healthy consumption norm (H1), the misperceived norms are calculated by subtracting the actual mean number of fruits, snacks and drinks from the perceived actual mean number. Next to exploring whether factors play a role in the susceptibility to social norms in a qualitative manner, we will test whether norm (mis)perceptions can be predicted by factors related to uncertainty reduction or affiliation purposes (e.g., measures 5.1–5.3) using regression analyses (H2). We will only include data in the analyses from participants who completed the survey and did not withdraw their assent. Categorical variables are (dummy) coded as whole numbers. Statistical analysis is performed using SPSS Statistics 23 and R (2013). Statistical significance is considered at the *p* < 0.05 level.

## Study 2 – Online Experimental Study of Social Norm-Based Messages on Food Choice: ‘Memory and Planning Performance of Pregnant Women’

### Stepwise Procedures

#### Design and Participants

The first part of Study 2 (Part 2A) has a 2 (pregnant vs. non-pregnant women) × 3 (poster condition) between-subjects design. In a poster memory task, pregnant- and non-pregnant women are exposed to either a descriptive or an injunctive norm-based message or a message unrelated to eating or social norms (i.e., control condition). The memory task is followed by a planning task in which participants make food choices (i.e., dependent variable). Participants are assigned randomly to one of the experimental conditions. The order at which participants are exposed to the posters in the memory task is not randomized, because the first posters are used as memory practice trials. In order to avoid effects of order or sequences, the food choice pictures in the planning task are presented randomly and the snacks and drinks picture blocks are counterbalanced. Part 2B examines social normative predictors and moderating variables among pregnant and non-pregnant sample separately.

Study 2 takes place between May and September 2018 and has the same inclusion criteria as Study 1. We check whether participants participated in Study 1 and if they did, they will be excluded from Study 2 due to the fact that they are already informed about the general objective of our study. Recruitment of pregnant participants will also follow the same procedure as in Study 1, while non-pregnant females will be recruited by convenience sampling at similar locations (e.g., at yoga classes or Internet fora). Before participating in the online study, participants will provide informed consent.

Power calculations (G^∗^Power 3.1.9.2.) are based on the design of Study Part 2B, requiring more participants than Study Part 2A by conducting the analyses for two samples separately (i.e., pregnant and non-pregnant women). To detect a medium to large effect size using multiple linear regression (f^2^ = 0.15; power 0.95, *p* = 0.05) and estimated with seven potential predictors (e.g., hunger, liking of experiment, pregnancy duration, poster condition, self-esteem, health benefit, and fear of negative evaluation), approximately 74 participants are needed. Taking into account an attrition rate of 15–20%, this results in the recruitment of 90 pregnant and non-pregnant women (*N* = 180). Consequently, we aim to recruit at least 15 pregnant and 15 non-pregnant women per country.

#### Cover Story and Experiment

Participants are delivered a cover story to conceal the actual aim of the study. Participants are told that they are participating in a study called ‘Memory and Planning Performance of Pregnant and Non-Pregnant Women.’ The experimental study consists of three parts. After providing assent and filling in demographic information, participants complete a memory task. In the memory task participants are asked to memorize specific details of posters (e.g., pictures, colors, and text). One at a time, participants are exposed to four posters, three of which are bogus posters to conceal the actual aim of the study. The real stimulus displays a norm-based message (participants in the control group are exposed to a poster unrelated to social norms or food). After each poster, participants have to describe what they remember and answer specific questions about the poster. This procedure ensures that participants pay attention to the stimulus material and their recall of the message is used as a manipulation check.

The second part of the study is the planning task in which participants have to plan ahead their daily activities (‘*Plan your day tomorrow*’) by choosing pictures displaying themes such as clothing, activities and food and drinks they will wear, perform, and consume on the next day, respectively. Thereby, we aim to assess food choice after exposure to the poster message while concealing the actual aim of the study. Next, participants are asked to memorize the pictures of part one again (used as additional manipulation check). In the final part of the study, they answer a questionnaire concerning moderator variables selected from the results of Study 1, and mixed with bogus items. Participants are asked to guess the aim of the study before they are debriefed, and they will have the opportunity to withdraw before ending the study.

### Materials and Equipment

#### Stimulus Material

During the memory task, one of the four posters displays a social-norm based message with either a descriptive or injunctive norm. The descriptive norm provides information about what the majority of others consume and the injunctive norm about what most other people think (non) pregnant women should consume. The control condition involves a message unrelated to social norms, health and food.

#### Measures

##### Food choice

The dependent variable consists of the participants’ selected food choice items and quantity. During the planning task, a combination of 32 pictures of healthy (e.g., apples, snack tomatoes, and dried fruit) and unhealthy snacks (e.g., cookies, sausages, and savory pastry) in small or large portions are shown. Likewise, there is a combination of 20 pictures of healthy (e.g., water and tea) and unhealthy (e.g., energy drink and chocolate milk) drinks, presented in 1 or 3 glasses. Food choice is calculated using kilocalories (kcal) for unhealthy and healthy foods and drinks separately.

##### Demographics, Pregnancy Related, and Moderator Variables

Administered demographic and pregnancy related measures follow the procedure described for Study 1. Moderator variables are selected based on the outcomes of Study 1 (as presented in the Method section of Study 1).

##### Manipulation Check

Recall, perception and credibility of normative and non-normative information are assessed in the experimental and control conditions (Stok et al., 2012). Recall of the message is assessed by checking whether participants memorized the norm message. Perception of the norm is checked by asking whether they thought that the percentage of other people referred to in the poster was low or high, and credibility of the normative statement is assessed by asking whether they found the norm to be credible [answering on a six-point scale ranging from ‘*Not at all*’ (1) to ‘*Very much*’ (6)]. In addition, the software program Qualtrics measures how long participants watched the posters (in seconds) to check whether participants actually paid attention to the poster.

##### Control Variables

Control variables that have been shown to affect food choice or the social norm manipulation are assessed, such as hunger, time of day, liking of the poster, etc. (Bevelander et al., 2012). These are measured at the end of the experiment to conceal the real aim of the study. Participants’ subjective hunger state and liking of the poster are assessed by a slider on a Visual Analog Scale (VAS) ranging from 0 to 100 (e.g., ‘*Not hungry at all*’ to ‘*Very hungry*’). It is also registered when participants engage in the experiment, because time of the day can affect participants’ food choice (i.e., afternoons are more commonly snack times than mornings) (Cross et al., 1994).

### Proposed Analysis and Anticipated Results

Data is screened to identify and remove outlying values as well as participants that terminated their participation early or who guessed the study’s aim. Scale reliability and construct validity is assessed by factor analysis according to standard procedures. Randomization checks are performed testing for differences between the poster conditions on demographic variables and potential control variables (e.g., age and hunger) by use of one-factor analysis of variance (ANOVA). Randomization is successful when there are no significant differences between conditions. In case there is a significant difference, the variable is added as a control variable in the main analyses. In a similar manner, manipulation checks are performed to check whether all participants were exposed to the poster and the cover story was delivered successfully. Participants who do not recall the poster message are excluded from the analysis. If there is a difference between poster conditions on credibility, this variable is added as a control variable in the main analysis. Next, Spearman’s rank and Pearson’s correlations are calculated for the demographic variables, control variables and the outcome measures (un)healthy food choice (in kcal) to determine which variables have to be included in the main model as covariates.

For Study Part 2A, multivariate analysis of (co)variance (MAN(C)OVA) is performed by testing a main effect of poster conditions on food choice and an interaction with pregnant and non-pregnant women. Pairwise comparisons are carried out with Bonferroni correction to determine significant differences between the experimental conditions. These analyses provide more insights into our hypotheses (H3&H4) that pregnant women exposed to an injunctive norm-based message would have a higher unhealthy intake than pregnant women exposed to a descriptive norm or control condition, and that this effect is stronger in non-pregnant women. We also expect that a descriptive norm message has a greater effect on both pregnant and non-pregnant women’s consumption behavior compared to a control condition. The analyses of Study Part 2B further scrutinize the moderating role of determinants relating to affiliation purposes and uncertainty reduction within the pregnant and non-pregnant sample separately. No hypotheses are formed because this is the first study that explores the role of these potential moderating factors. Statistical analysis is performed using SPSS Statistics 23 with a significance level of *p* < 0.05.

## Limitations

Exploratory studies go along with a number of limitations. First, the online studies use non-probability sampling methods (i.e., purposive as well as convenience sampling), possibly resulting in a sampling bias (Etikan et al., 2016). For example, by an over-representation of pregnant women who are concerned about their own and child’s health (i.e., women who participate in yoga lessons or search for information on online parenting forums) or an under-presentation of women who are digital illiterate or live in a remote area without appropriate Internet access. To strive for a heterogeneous and representative sample of pregnant women, the studies will be advertised at locations where pregnant women go (e.g., midwives or medical offices and diverse Internet fora). Further, this project is exploratory and the goal is to generate and refine hypotheses which will be particularly valuable for the directions of future research. Second, this project administered self-reported recall and predictions of food intake and choice only. Future research would profit a great deal from including real eating behavior using, for example, daily reports in a diary study or an experimental setup in a natural environment. Third, there may be socio-cultural differences between some of the European countries that affect women’s susceptibility to social norms and their eating behavior. For example, it is possible that social norms and expectations are more stringent across Southern than Northern European areas (Heinrichs et al., 2006; Gelfand et al., 2011). That is, in some countries there may be a greater focus on close family relationships and the community that could magnify the effect on normative social behavior (e.g., Spain and Cyprus) (Campos et al., 2008) whereas this may be less pronounced in other individualistic countries such as United Kingdom and Netherlands (Gelfand et al., 2011). In addition, some literature on eating patterns suggest differences across North-European, Western and Mediterranean cultures (Wolff and Wolff, 1995; Cuco et al., 2006; Northstone et al., 2008; Crozier et al., 2009) whereas other research shows that there is a progressive narrowing of differences in dietary patterns across Northern and Southern European countries (Naska et al., 2005). Therefore, we will explore whether there are socio-cultural differences between countries. If we find differences in Study 1, we take appropriate measures by, for example, dummy coding Northern and Southern European countries in our analyses. In addition, we will adjust the sample size of Study 2 to avoid the experiment from being underpowered by performing new power analyses. This will improve the generalizability of our findings across the different countries. Finally, the use of self-reported data often results in dealing with social desirable answers and participation bias. To prevent this, the aim of the study is explicitly stated and the anonymous data handling is stressed in Study 1. In addition, the cover story used in Study 2 limits social desirable answers.

## Conclusion

This project aims to fill a research gap by broadening the existing scope of research into social norms. Combining exploratory research with research methodology used previously in social norm research among pregnant and non-pregnant populations enables comparing pregnant with non-pregnant women. This contributes to more knowledge about how women perceive guidelines from their social environment, which underlying mechanisms play a role and whether social norms can be used to stimulate healthy eating. Moreover, cultural aspects that co-determine which social norms and guidelines exist are taken into account. Social norm campaigns have shown to successfully change misperceived norms (DeJong et al., 2006; LaBrie et al., 2008) and promote healthier eating (Robinson et al., 2014a; Stok et al., 2014) by removing uncertainty about how to behave. The project’s findings will help to develop and design effective messages for interventions in promoting healthy eating behavior specifically targeted to pregnant women among different European societies.

## Ethics Statement

Both studies were conducted according to the principles laid down in the Declaration of Helsinki (World Medical Association, 2013). The studies were approved by the Ethics Committee of the Faculty of Social Sciences (ECSW-2017-012) of the Radboud University, Nijmegen, Netherlands. The study procedure, materials, and consent forms were reviewed in accordance to ethical guidelines ‘Code of Ethics for the Social and Behavioural Sciences’ and ethical standards in Dutch as well as European context [EU; General Data Protection Regulation (GDPR)]. Both studies involved online anonymous voluntary data collection procedures among adults without any expected adverse events. Each participant provided active consent for participation after having received information about the aim and procedures of the study. Although Study 2 used a cover story to recruit participants, it had a debriefing at the end of the online experiment. All participants were also given the opportunity to ask questions to the researchers and withdraw their assent during and at the end of the studies.

## Author Contributions

KB conceived and initially designed and wrote the protocol. KH, CK, IS, A-LT, and MT contributed equally to the research design, wrote parts of the manuscript, and provided important critical feedback when revising the manuscript. All co-authors are listed in alphabetical order. All authors approved the final version of the manuscript.

## Conflict of Interest Statement

The authors declare that the research was conducted in the absence of any commercial or financial relationships that could be construed as a potential conflict of interest.
